# Summary of the Transactions of the College of Physicians of Philadelphia, from February 6, 1849, to April 3, 1849, Inclusive

**Published:** 1849-09

**Authors:** 


ART. II.—*Summary of the Transactions of the College of Physicians of Philadelphia, from February* 6, 1849, *to April* 3, 1849, *inclusive.*

The College of Physicians of Philadelphia, is an association holding stated monthly meetings for the reception of formal reports on scientific subjects relating to Medical science, for verbal communications of facts and observations, and for discussion of any topics which may be presented. A summary of transactions is issued quarterly, always containing much that is interesting and valuable. We regard this as one of the most useful of the periodical publications of our country, and it seems to us much to be desired that associations in other places should pursue a similar plan. Valuable contributions to medical knowledge and literature might in this way be secured. We have, on former occasions, called the attention of our readers to the useful labors of this association, and expressed the same sentiments. The number of the Transactions now before us contains an elaborate report by Dr. J. Wilson Moore, exhibiting complete meteorological observations for each month in the year, and, in connection therewith, the mortality of diseases for every month. It embraces, also, a table showing the mortality in each year for thirteen years. Next follows a paper by Professor Jackson, communicating observations on Hydrophobia, with cases. Next, a paper by Dr. Bond, giving an "account of two cases of retroversion of the uterus, with a description of a new instrument for its restoration and some observations on the displacement of the organ." Then follow remarks and discussions by different writers on various topics. From the latter, we proceed to make large quotations, believing that they will be perused with satisfaction, and presuming that the "Transactions" circulate to a limited extent among our readers. We would again express our obligations, individually, to the College of Physicians for their valuable publication, and, from the extracts which we have made from its pages, not only now, but on former occasions, we doubt not our readers are disposed to add their thanks to ours.

"Dr. CONDIE remarked, that since the last meeting of the College, a case had fallen under his notice, which, though of little interest in itself, would serve as another fact to be added to those already accumulated in reference to the effects of etherization.

Ahout 11 o'clock, on the evening of the 7th of February last, he was requested to meet Dr. Scoffin, in consultation, in a case of great emergency. On arriving at the house of the patient, he learned that Mrs. H., a lady of about 27 years of age, of short stature and dark complexion, had been taken in labour about three hours previously, and that simultaneously with the occurrence of the parturient pains, she had been seized with convulsions; a violent paroxysm of which was induced upon each return of the pains. The lady was the mother of one child, which was born after a somewhat lingering labour, but unattended with the slightest accident. For many weeks she has experienced a good deal of anxiety in consequence of an anasarcous swelling which had commenced in her lower extremities, but finally extended over her whole body. The intumescence was very considerable; her face was bloated, and her lower extremities, especially, enormously distended.

On an examination per vaginam, the head of the child was found to be high up, and the os uteri very tense, and but little dilated. Instrumental delivery was of course out of the question.

The convulsions were most violent, and the safety of the patient demanded prompt relief. The pulse being full and somewhat tense, I recommended that twenty ounces of blood should be taken away, and the patient then placed under the influence of ether. The anæsthetic effects of the ether were very quickly induced, and although the patient did not lose entirely her consciousness, she experienced no pain, nor were the convulsions repeated, so long as she remained under the influence of the ether. Labour, nevertheless, went on regularly; an increased secretion of mucus occurred in the vagina, the os uteri lost its rigidity and readily dilated, and in less than two hours the lady was delivered of twins, the first, a vertex presentation, was born alive; the second, a footling presentation, was dead born. At the termination of the labour, the inhalation of the ether was discontinued, and very soon the convulsions returned with nearly the same violence as before. They were finally subdued by cups to the head, a blister to the back of the neck, sinapisms to the extremities, and active purgative enemata. The lady and her child are both doing well.

The ether appeared, in this case, to be beneficial by suspending the convulsions until the labour was terminated, which latter it appeared, also, to accelerate.

Dr. Griscom stated that since the last meeting of the College, he had resorted to etherization in the case of an unmarried lady who had been for a long time affected with irregular menstruation, and occasional attacks of pain resembling the most violent attacks of dysmenhorrœa, though the patient was not at the time menstruating. The sufferings during these attacks were most intense; in one case the pain inducing a state of unconsciousness. The ether was not administered to such an extent as to destroy the consciousness of the patient; it was repeated about every hour until the pain ceased. With the effects of etherization in this case, Dr. G. stated that he was much pleased.

He had employed the remedy this day in a case of labour, with the most satisfactory results. The sufferings of the lady in her former labours had been very intense, and almost without an interval of ease for many hours. The commencement of her labour to-day presented indications that it would be as protracted and attended with pains of equal intensity. The ether was administered to an extent sufficient to suspend her sufferings without inducing a state of unconsciousness. Independently of the relief of the pain procured by etherization, the labour was very considerably shortened by it, being terminated within two hours, instead of continuing for six hours as formerly.

Dr. Bond stated, that about ten days ago he was called to see an elderly female, who had enjoyed all her life uncommonly good health; never, within her recollection, having any ailment requiring the attendance of a Physician. Some time previously to his visit, she had been attacked with neuralgia of the side of the head, extending around the ear. She at first believed that it would go off without medical advice, but the pain increasing in intensity, Dr. Bond was sent for. He directed a portion of muslin cut in such a shape as to be applied directly upon the affected parts, to be wet with chloroform, and after its application to be covered with oiled silk to prevent evaporation. The relief was almost instantaneous; the chloroform was repeated at short intervals,—and the pain was very soon entirely removed.

Dr. Griscom remarked that he has used the chloroform externally with very happy results, but confesses that he is somewhat fearful of its effects when exhibited by inhalation.

Dr. Bond is perfectly aware that it is a very common opinion that the ether is a much safer anæsthetic agent, for internal exhibition, than the chloroform, but he believes this to be an error. He considers the chloroform the safest of the two. Ether produces, frequently, before its anæsthetic effects are manifested, a state of very considerable excitement, but nothing of the kind, so far as his experience goes, is caused by chloroform. Dr. B. is inclined to think, that the real cause of danger from the exhibition of chloroform, is the improper manner in which it is frequently exhibited. Being the most powerful agent of the two, the greater is the importance of caution being observed in its inhalation, and that it be well diluted with atmospheric air.

Dr. Wood remarked, that to him, the action upon the system of the chloroform and ether, appears to be very different; the first being directly sedative, while the sedative effect of the ether is secondary, its first action being that of an excitant. Hence the danger from the use of the chloroform is most liable to be immediate. If the patient pass safely through the first effects of the article, there is little danger of any organic lesion resulting. Hence chloroform is a less doubtful remedy in cases in which there is any tendency to local inflammation or local congestion than the ether.

Dr. Parrish has used chloroform, locally, in the supra-orbitary pains incident to cases of rheumatic ophthalmia, and in the deep seated pains, common in inflammatory affections of the eye, and has derived from it the most striking relief. In these cases very large doses of opium are of little avail.

In a case of syphilitic ulceration at the root of one of the finger nails, attended with intense suffering, at night, from pain, extending up the arm, and depriving the patient of repose, the local application of chloroform produced very decided relief, enabling the patient to pass very comfortable nights. Opium and various other remedies had been used, and without producing any decided or permanent relief.

Dr. Coates inquired of Dr. Hays, whether the journals present any cases in which the local application of either of the anæsthetic agents has been resorted to as a means of relieving pain previously to surgical operations? It is very certain that the external application of chloroform will benumb the sensibility of a part, and if, upon trial, it shall be found that the effect is sufficiently permanent to enable a surgical operation to be performed without pain, the danger resulting from the internal use of chloroform could be avoided. He is aware that by some persons pain is considered to be a useful stimulant in operations, by which the recovery from them is rendered more prompt and certain. To this doctrine he is not prepared to subscribe; he believes pain to be an evil, and that, when severe, it exhausts the powers of life, and may even produce death! Hence he considers every rational means for its prevention to be worthy the attention of the Surgeon.

Dr. Hays replied, that the local application of chloroform, as the gentleman is no doubt aware, has been suggested by Dr. Simpson for the production of anæsthesia in surgical operations, and has been actually employed for that purpose by Dr. Nunnelly. The local application of cold, has also been suggested by Dr. Arnott, as an effectual means of destroying pain in operations.

Dr. H. stated that the gentleman whose case was mentioned by him, at a late meeting of the College, has since had another attack of the neuralgia in his lower extremity; the application of the chloroform was again resorted to, and it afforded relief, but not quite in so decided a degree as it did at first. In another case of violent neuralgia in which he has repeatedly employed the remedy, locally, the patient says that the pain is always arrested by it.

Dr. Coates proposed the use of the tincture of aconite as an anæsthetic agent; it has the power of benumbing the sensibility of the surface upon which it is applied, and he believes that to relieve pain, it might be very advantageously used, both internally and externally. It is true that the local application of this article would not be prudent upon parts about to be divided by the knife. He believes, however, that it may be used internally with perfect safety, to an extent sufficient to ensure its anæsthetic effects.

Dr. Hays remarked, that the aconite is perfectly safe as an external application, but nothing short of a saturated tincture will have the effect of controlling pain. He has often used it with benefit in cases of neuralgia. An ointment, composed of one or two grains of aconitina, rubbed up with a drachm or two of lard, is far more effectual than the tincture of aconite. It produces in the parts upon which it is applied, a sensation of coldness succeeded by numbness. He attended a lady from North Carolina affected with violent neuralgia of the supra-orbitary branch of the fifth pair, in which the application of a portion of the ointment, of the size of a small pea, never failed to produce prompt relief. It acts as a direct sedative. A strong solution of the cyanuret of potassium will produce, also, anæsthesia.

Dr. Jackson can testify to the anæsthetic effects of the saturated tincture of aconite when applied externally. He himself suffered a short time since from an attack of violent neuralgia of the side of the face, extending to the eye; a portion of the tincture of aconite was rubbed over the seat of the neuralgia, and in fifteen or twenty minutes the pain entirely ceased. The parts felt benumbed, and the skin, as it were, indurated. After continuing the use of the remedy for five or six days, the disease entirely disappeared.

He has since tried it in a case of tic doloureux of the jaw, but unsuccessfully.

Dr. Page has employed, frequently, the ointment of veratrine externally, for the relief of neuralgia. The topical application of all the active narcotic poisons, as a means of relieving pain, has long been known. But the question is, can either of them be employed in this manner, successfully, to prevent the pain of surgical operations? Whether any of them will answer, when thus employed, to effect this important end, he is unaware. If, upon trial, they should be found to succeed, a most important end will be gained, and the objections to the production of anæsthesia by the internal use of ether and chloroform, removed.

Dr. Coates related a case of intense pain in the temple which was relieved by the remedy; the face feeling like parchment.

Dr. Parrish has known a poultice composed of the green or even dried leaves of the stramonium, to act quite as effectually as chloroform in relieving the pain occurring after operations upon the eye.

Dr. Bond has frequently used the ointment of stramonium in neuralgia with good effect.

Dr. Wood believes that the credit of suggesting the local use of chloroform as a means of relieving pain, is due to the physicians of this country. It was first preposed in Boston, by an anonymous writer, in the Medical and Surgical Journal of that city, and, was afterwards brought before the British Association, by Dr. Nunnelly, as a means likely to be useful in many cases; but the first instance in which he was aware that the local effects of chloroform were actually tested, for the relief of neuralgia, was in the case related by Dr. Hays, at the last meeting of the College.

Dr. J. F. Meigs inquired whether either of the Fellows could give him any information as to the effects of the seeds or root of the burdock in cutaneous affections. A practitioner, from the interior of the state, Dr. Riter, mentioned to him, recently, that he had succeeded in curing many cases of psoriasis by a saturated tincture of the seeds of the burdock. He had suffered, himself, for many years, from psoriasis upon his legs; at the suggestion of a non-professional acquaintance, he was induced to make a trial of the burdock. He used a table spoonful three times a day, for several months, when an entire cure of the disease was effected.

Dr. Page stated that the narrow leafed dock is a very common remedy in the South in cutaneous diseases and ulcers.

D·. Norris remarked that the yellow dock is used constantly in the New York Hospital as a remedy in cutaneous diseases.

Dr. Moreton Stille stated, that in a case of psoriasis, admitted into the Pennsylvania Hospital, in which the disease covered the entire scalp, and with large patches upon the limbs and different parts of the body, three drops of Fowler's solution were administered, three times a day, with half a pint of decoction of dulcamara daily, the parts affected being, at the same time, bathed with a decoction of flaxseed, while the tar ointment was applied to one arm. The scales soon commenced to fall off, and the eruption to assume a better appearance, especially upon the arm to which the ointment had been applied. The iodide of sulphur was now applied to the other arm: this appeared to produce a much more rapid effect than the tar ointment. It was therefore applied to the other parts of the body. The patient has been under treatment six weeks, and exhibits no trace of the psoriasis, excepting a discoloration ef the parts it occupied. His general health is good. To the scalp, which was covered with a thick crust, the tar ointment was applied for two weeks, and under its use the entire removal of the disease took place.

Dr. Jackson has been in the habit of employing an ointment of sulphuric acid in cases of psoriasis. Under its use the disease, in general, began speedily to improve, and by continuing steadily the ointment, got well, finally, without the necessity of resorting to other remedies.

Dr. Parrish relatod a case of the fracture of the superior maxillary bone, extending to the antrum, but not to the alveola. It occurred in a young man, who, coming down stairs, with a bundle in his arms, fell with his face against a box. A concussion of the brain was produced; on his recovery from the effects of this, a slight deformity of one side of the face, consisting in a flattening of the cheek, was discovered. It was only apparent when looking the patient full in the face; it was attended with no pain, and was so trivial as to escape, for some time, the observation of his friends. A casual remark of an acquaintance induced him to place his finger upon the cheek, when he felt a slight prominence. Dr. Parrish was immediately consulted, who considered that nothing could be done to replace the bone; in this opinion Dr. Barton, who saw the case with him, coincided. No inconvenience has ensued from the fracture beyond the very slight deformity, which remains.

Dr. Stille remarked, that a similar case had been brought into the Pennsylvania Hospital last summer. The patient had been struck on the side of the face by a door which was suddenly blown to by the wind. A slight concussion of the brain immediately ensued, upon recovering from which, a fracture of the upper maxillary bone, extending vertically, but not to the alveola, could be traced by the finger. No inconvenience resulted, excepting a slight, scarcely perceptible deformity.

Dr. Page called the attention of the College to the lupuline, as a means of controlling the painful erections occurring in venereal cases. He has employed it now for two years, and has found it a better and more effectual remedy than any other he has tried. He gives it in the dose of from five to ten grains, and has never known an instance in which the second dose did not succeed in subduing the painful erections, so troublesome in cases of gonorrhœa. It does not cause the head-ache, constipation, and other unpleasant effects consequent upon the use of camphor and opium. He has found the remedy useful also in cases of involuntary seminal emissions. It will not cure the disease, but prevents the discharges, so long as the patient remains under its influence.

Dr. Bond stated that he had employed the tincture of iodine for the removal of pruritus vaginæ; in a number of instances it has caused prompt relief. It should be applied daily to the parts. It produces, at first, a slight glow, which soon goes off, but never any considerable smarting.

Dr. Griscom has employed the cod liver oil, lately, in numerous instances, and cannot but think that in some cases of incipient phthisis it has had a beneficial effect.

Dr. Hays remarked, that he had seen, within a few years, some curious cases of exalted sensibility of the retina, from a cause which he believes has not been suspected of such an effect, viz: irritation of the dental branch of the fifth pair of nerves. These cases much interested him, and if the College had nothing better to occupy their attention, he would present a verbal sketch of them; he had not prepared any written history of the cases, having no previous intention of submitting them at this meeting.

The first case occurred in a gentleman, the Cashier of a Bank in North Carolina. At the great fire in Wilmington, he had suffered considerable fatigue and exposure in endeavoring to save the books and papers of the Bank, and had, subsequently, severely tried his eyes in arranging the documents which were rescued from the flames. He soon experienced great intolerance of light. For this he was treated by the physicians in his vicinity, but with only temporary relief. He subsequently visited Virginia and Raleigh, North Carolina, for medical advice; but from none of the remedies or plans of treatment employed in his case, did he experience the slightest permanent benefit; on the contrary, the intolerance of light increased to such a degree as to render exposure to light perfect torture. Dr. Hays was written to. Believing the case to be one for which it was not possible to prescribe judiciously until he was enabled to make a thorough examination of it, he requested that the gentleman should be brought on to Philadelphia; but his friends, in reply, suggested that this would scarcely be possible, in consequence of the excessive photophobia under which the patient labored, rendering the slightest degree of light intolerable. Dr. Hays suggested that the eyes should be entirely defended from the access of light by covering them with a mask of wadded silk. This suggestion was adopted. When the gentleman reached the city, Dr. H. found him laboring under the most aggravated degree of photophobia. In a room so perfectly dark that the Doctor was unable to see any object whatever, to the patient, the light reflected from his own hands, was intolerable, and that from his shirt bosom caused so much suffering that he was obliged to keep the latter constantly covered. The colored nurse, whom he had brought on to attend upon him, happening to enter the darkened room, the light from a white apron she wore produced so much suffering to the patient, that he flew into a violent passion at the poor creature, and threatened punishment should she ever enter his presence again similarly attired. So exalted was the sensibility of the retina, that in the darkened room, where Dr. H. could not see his hand held up before him, the patient was able to distinguish the objects aronnd him, even the figures in the carpet. He was, at length, persuaded to submit to an examination of his eyes, which he bore with great fortitude. Dr. Hays found scarcely a trace of inflammation of the eyes or of any other apparent disease. The stomach of the patient was somewhat deranged. This being remedied without relief to the photophobia, the Doctor was induced to seek for some other source of irritation, and, after reflection, he was induced to suspect that the teeth, several of which were defective, but not painful, might be the source of the evil. At his suggestion a couple were extracted, but without causing any diminution of the intolerance of light. After some eight or ten days, Dr. H. examined the patient's mouth himself, and upon striking one of the lateral upper incisors nearest to the eye most affected, with a key, the patient winced as from pain, and stated that he had often experienced a disagreeable sensation to proceed from that tooth. The tooth was extracted; with the loss of the tooth, a most disagreeable gnawing or pinching sensation at the back of the eye, which had previously tormented him, ceased. At the root of the tooth there was found a large abscess, while the periosteum of the alveola was thickened. From this time the morbid sensibility of the eyes rapidly diminished, and the patient was soon after sufficiently recovered to return home and resume his duties as Cashier of the Bank. When Dr. Hays last heard from him, which was last summer, after an interval of nearly six years, he was perfectly well, having had no return of the photophobia.

The next case was one which Dr. H. saw last fall, in consultation with his friend Dr. Ashmead, in a Spanish gentleman, who had suffered two years previously from a slight attack of iritis. On recovering from this, he experienced, whenever he attempted to read, a peculiar uneasiness in his eyes. For this, he consulted Dr. Ashmead, who advised a voyage to New Orleans; finding no diminution in the affection of the eye, he proceeded from New Orleans to Cuba. The uneasiness still continuing unrelieved, he returned last fall to Philadelphia. Dr. H. now saw him with Dr. Ashmead. Upon examining the eyes they were found to be without any trace of inflammation or other apparent disease. Still, whenever the patient attempted to read, the same uneasiness, which had now continued for some eighteen months, was experienced. Judging from the circumstances of the case first detailed, Dr. H. was led to suspect that the source of the affection of the eye in the present case might be the same; he accordingly examined the patient's teeth, from which, according to his account, he had experienced no suffering. Finding some of the teeth diseased, the Doctor directed them to be extracted. One only was taken out, when the patient's courage failed him, and no relief was afforded to the affection of the eye. Subsequently, another tooth was extracted, at the root of which an abscess was found to exist. The patient now declared himself entirely relieved from the uneasy sensation he had so long experienced in his eyes on attempting to read. He has since continued perfectly well; now eighteen months.

Another case occurred in a lady, marked by the same intolerance of light as in the case first described; in which Dr. Hays had every reason to believe that the morbid sensibility of the retina was produced by irritation of the dental branch of the fifth pair of nerves. On examining the patient's teeth, several were found diseased. Five were extracted; at the roots of three of them there existed an abscess. The gums continued sore for some time; but the photophobia was considerably relieved. The patient passed from under the care of Dr. Hays into that of another physician. He heard subsequently that she had entirely recovered; and as no other treatment had been resorted to in her case, excepting covering the eye with a single slip of linen, moistened with water, and a shower bath, he believes that he is not in error in referring the cure in this case to the extraction of the diseased teeth.

The last case he shall refer to, was that of the daughter of a physician of Wisconsin. She had been subject to frequent severe attacks of inflammation of the eyes. In July last she suffered from one of these attacks, which was followed by excessive intolerance of light, that no remedy employed by her father seemed in the least to relieve. She was taken to Washington City, where she was pronounced to be incurable by several physicians to whom her case was made known. She was then brought on to Philadelphia, and placed under the care of Dr. H. At his first visit so great was the intolerance of light, that no satisfactory examination of the eyes could be made. From the imperfect view Dr. H. obtained of them, he ascertained that they were somewhat inflamed, and that a slight opacity of the cornea existed. Several of the young lady's teeth were decayed. Dr. H. directed two to be extracted, but without much relief to the morbid sensibility of the eyes. In a week or ten days two or more of the teeth were extracted from the upper jaw. After a few days the intolerance of light was greatly diminished. The lids were painted with tincture of iodine, and treatment directed, calculated for the relief of the vascularity and opacity of the cornea, and in three months, she so far recovered as to be able to read in a diamond print Bible, and bear an ordinary degree of light, when she left for home. Since then the doctor has hot heard from her.

If he is not mistaken in his view of these cases, Dr. Hays believes they very conclusively show that intolerance of light, and other uneasy sensations of the eye, may originate in an irritation of the dental branch of the fifth pair of nerves, resulting from diseased teeth—and the cure of which can only be effected by the removal of the latter. The first two cases appear to prove this incontestibly; the symptoms, after resisting all the other means of cure adopted, promptly disappeared upon the extraction of one or more decayed teeth, thus showing the due relation between the effect and its cause. In the last described case, though the intolerance of light was, no doubt, owing to the inflammation of the eye itself, still he conceives that it was in part, at least, kept up by irritation of the dental branch of the fifth pair, from decayed teeth.

Dr. A. Stille inquired what had been the treatment employed in these cases, previously to the extraction of the teeth;—whether any of the anæsthetic or narcotic remedies had been resorted to, and with what effect?

Dr. Hays replied, that in the first case, bleeding, leeching, and purging, had been tried; by these the disease was, at first, somewhat relieved, for a time, but then returned with redoubled violence. The tincture of aconite, veratrine, aconitina, had severally been applied around the eye, by him, for several weeks before the real source of the disease was suspected, but with little or no benefit. So far as regards the intolerance of light, the failure of these remedies may, with propriety, be said to have been complete, in the first case. The lady from Wisconsin had been under the care of her father, from July to November, and although a skillful physician, and accustomed to the treatment of affections of the eye, she assured Dr. H. that she received no benefit from any of the remedial measures employed in her case.

Dr. Ashmead remarked, that in the second case no remedy or plan of treatment resorted to, previously to the extraction of the teeth—and almost every thing else had been tried—was productive of the least benefit.

Dr. A. Stille observed, that the object of his question had been, to ascertain whether the remedies, employed anterior to the extraction of the teeth, had been followed by any beneficial result, or had failed entirely to give relief. As they were entirely unproductive of good, we may, in future cases of nervous affection of the eye, when a similar failure in our remedies is experienced, be led to expect that some such exciting cause, as in the cases just detailed by Dr. Hays, exists, and by discovering this, and, if possible, removing it, be enabled to cure the affection of the eye.

Dr. Parrish remarked that the subject introduced this evening to the notice of the College, by Dr. Hays, was a most interesting and important one. The cases detailed had been to him most instructive, and he should, certainly, hereafter, never neglect a cautious examination for the detection of any source of irritation from decayed teeth, in the cases of exalted sensibility of the cornea, which come under his notice.

His object in rising, now, was to point out the fact, that there frequently occur cases of ophthalmic disease in which intolerance of light is kept up by the very means resorted to to cure it; the careful exclusion of the eyes from light, and the confinement of the patient in a darkened room for many weeks, and even months. From the prolonged want of exercise and fresh air, in such cases, the energies of the system become so reduced, and the nervous sensibility so morbidly exalted, that the patients are able to discern the minutest objects in a room which appears perfectly dark to the healthy eye—while, at the same time, they cannot tolerate, without intense suffering, the slightest degree of light.

These cases are generally those of scrofulous ophthalmia, in which the seclusion and confinement are doubly prejudicial—augmenting the disease, while they induce an exalted sensibility of the eyes. At the Wills' Hospital it is the habit, in patients who have been thus injudiciously treated previously to their reception, to gradually accustom the eyes to the light, and with the best effect. In a most interesting case, which was brought to the institution from New Jersey—a child five or six years old, who had been excluded from the light for a long period, we encouraged an exposure of the eyes, gradually, to the light out of doors. The third day afterwards the child was brought to us with its head up, and its eyes wide open. By the use of tonic remedies; iron, and quinine, the salt bath, and nourishing diet, the patient was soon afterwards restored to health.

Dr. Parrish saw a case with Dr. Griscom. The patient, a child between four and five years of age, was affected with scrofulous inflammation of the eyes, with great intolerance of light, which had lasted for some eight, nine, or ten months. The patient had been closely confined to a dark room. He recommended a gradual exposure to the open air, with the same tonic treatment as in the former case. In a short time the eyes were entirely well, with the exception only of a slight opacity of the cornea.

At the Wills' Hospital, a man was admitted with ulcer of the cornea, attended with great intolerance of light. He was advised to go to sea, and accordingly entered on board a vessel bound to Cuba. He returned perfectly cured, notwithstanding the exposure and fatigue to which he had been subjected. On leaving Cuba, he had gone to Florida and entered the army, performing all the duties of a common soldier. He stated that his eyes were improved the moment he got out to sea.

Dr. Hays remarked, that the cases just related by Dr. Parrish were not precisely similar to those just described by him. He can, however, add his testimony in favor of the beneficial effects of fresh air and open exposure, particularly on the sea shore, in the photophobia of scrofulous ophthalmia. He had a case in a child between five and six years of age, in which the photophobia was so considerable that the patient walked with half closed eyelids and depressed head, even when passing through a darkened entry. After a preparatory treatment, consisting, chiefly, of a course of mild purgatives, he advised the child to be taken down to Cape May. The parents objected, fearing, as she could not endure to open her eyes in the obscurity of a darkened room, the light of the day wouid cause her intolerable suffering; but as the doctor insisted upon her going, she was taken down. The evening she arrived there she ran about the sea shore, without making complaint of the light, next day she was able to do the same, and the photophobia soon disappeared entirely.

Professor Jackson observed, that, as in the cases detailed by Dr. Hays, the sensitive branch of the fifth pair going to the eye was affected by an irritation, originating in its sensitive branch distributed to the teeth, so in other cases, an irritation affecting the dental branch, will extend to other branches of the nerve than those of the eye. Thus, a gentleman solicited his advice in consequence of an intense pain he suffered, extending over the right side of the face and right ear. In the absence of febrile symptoms, or indications of local disease, the doctor suspected that the pain was the result of some defect in the teeth, causing irritation of the branch of the fifth pair of nerves distributed to the affected part. He inquired of the patient, whether he had any decayed teeth; but the reply was, no, I never have toothache or uneasiness about my teeth. Dr. Jackson examined the jaws, but could detect no unsound tooth, and a dentist of considerable skill was equally unsuccessful. Narcotic poultices, and other calming remedies were resorted to, but without producing any permanent relief. Mr. Townsend, the dentist, upon a careful examination of the patient's teeth, ascertained that there was one, which, when sounded, was slightly painful; this was extracted, and an abscess was discovered at its root. The disease of the face now quickly disappeared.

In other cases, again, it is the motor branches of the fifth pair in which the irritation is produced, and spasms of the muscles to which the irritated branch is distributed, are the consequence. A young lady was placed under his care affected with trismus. The teeth could be separated for a short distance, but in a short time the jaws became again partially closed. The lady's general health was good. By means of an instrument, which the professor had constructed, he succeeded in separating the jaws, so as to examine the teeth. They were all, to appearance, sound. Dr. Roper was now requested to make an examination of the teeth, and after some time, by means of a small hooked instrument, he detected a defect in the last molar tooth. It nad given the lady no pain or uneasiness which she could refer to that part. The tooth was, with much difficulty, extracted, and the trismus from that moment ceased.

Professor Jackson was consulted by Dr. Dickey, in a case of trismus, similar to the one just detailed. He suggested an examination of the teeth; one was found to be diseased, and was extracted. In two or three days the lady was entirely well.

Dr. Hays remarked, that he has met with many cases of severe neuralgia of the face which were cured by the extraction of decayed or otherwise diseased teeth, where there had been no complaint of tooth-ache. In the first four cases of photophobia, related by him this evening, the patients made no complaint of their teeth.

In the case of a lady who has labored under neuralgia of the supra and infra orbitary nerves, he has every reason to believe that the suffering originates from a defective front tooth; but he has been unable to induce a dentist to extract it, as the tooth is not decayed, and they fear to mar the looks of a beautiful woman. She still suffers from the disease. Temporary relief is obtained by the application of aconitina.

Dr. West has seen a case which illustrates the remarks of Professor Jackson. A gentleman was troubled with what he described as a pulsative pain in the eye; on examination, Dr. W. found the inconvenience complained of to be produced by a spasmodic twitching of the upper eyelid. The affection had lasted for some considerable time. Anodynes had been tried without effect. Upon examining the patient's mouth, Dr. West detected a diseased tooth, on the same side with the affected eyelid, and from the irritation thence resulting, he believed the spasmodic motion of the eyelid to result. The tooth was extracted, and the spasms ceased. They have not again returned, to the same extent as previously, but whenever the patient experiences any tenderness of the teeth, a slight twitching of the eyelid occurs."

